# 

**Published:** 1896-01

**Authors:** 


### CONTENTS.

ORIGINAL COMMUNICATIONS—

PNEUMONIA. By C. D. Hurt, M.D., Atlanta, Ga. .... 641

THE LOCAL APPLICATION OF COLD IN ACUTE PNEUMONIA. By Thomas J. Mays, A.M., M.D., Philadelphia, Pa. .... 647

THE DIAGNOSIS AND MEDICAL TREATMRNT OF INTESTINAL OBSTRUCTION. By R. F. Williams, M.D., Richmond, Va. .... 654

CURIOUS CASES OF ATROPIA POISONING. By N. C. Mitra, M.A., M.B., Bengal, India. 660

SOCIETY REPORTS—

ATLANTA SOCIETY OF MEDICINE .... 662

CORRESPONDENCE—

NEW YORK LETTER .... 671

THE MARION COUNTY (FLA.) MEDICAL SOCIETY .... 676

EDITORIAL—

THE STATUS OF THE ANTITOXIN .... 677

DR. THOMAS S. POWELL .... 682

THE JOURNAL'S PREMIUMS .... 683

READ THE ADVERTISEMENTS .... 684

SELECTIONS AND ABSTRACTS—

GENERAL MEDICINE AND THERAPEUTICS .... 685

EYE, EAR, NOSE AND THROAT .... 688

MISCELLANEOUS .... 691

MEDICAL ITEMS .... 704

BOOK REVIEWS .... 708

ADVERTISERS' INDEX TO ADVERTISEMENTS .... xiv

MEDICAL ITEMS—CONTINUED .... x, xi, xii, xiii

CLASSIFIED INDEX TO ADVERTISEMENTS .... xx

